# Differential Modulation of Cancellous and Cortical Distal Femur by Fructose and Natural Mineral-Rich Water Consumption in Ovariectomized Female Sprague Dawley Rats

**DOI:** 10.3390/nu11102316

**Published:** 2019-09-30

**Authors:** Cidália Pereira, David Guede, Cecília Durães, Inês Brandão, Nuno Silva, Emanuel Passos, Miguel Bernardes, Rosário Monteiro, Maria João Martins

**Affiliations:** 1 School of Health Sciences and CiTechCare - Centre for Innovative Care and Health Technology, Polytechnic of Leiria, 2411-901 Leiria, Portugal; cidalia.pereira@ipleiria.pt; 2 Trabeculae Technology Based Firm S.L., Technological Park of Galicia, 32900 Ourense, Spain; guede.d@icloud.com; 3 Ipatimup Institute of Molecular Pathology and Immunology of the University of Porto, 4200-135 Porto, Portugal; cduraes@ipatimup.pt; 4 i3S Instituto de Inovação e Investigação em Saúde, Universidade do Porto, 4200-135 Porto, Portugal; inesm.brandao@gmail.com (I.B.); rosariom@med.up.pt (R.M.); 5 Department of Biomedicine, Biochemistry Unit, Faculty of Medicine, University of Porto, 4200-319 Porto, Portugal; elpassos@hotmail.com; 6 Unidade I&D Cardiovascular do Porto, Faculdade de Medicina, Universidade do Porto, 4200-319 Porto, Portugal; nunosilva.box@gmail.com; 7 Department of Clinical Pathology, São João Hospital Centre EPE, 4200-319 Porto, Portugal; 8 CIAFEL, Research Centre in Physical Activity, Health and Leisure, Faculty of Sport, University of Porto, 4200-450 Porto, Portugal; 9 Department of Medicine, Faculty of Medicine, University of Porto, 4200-319 Porto, Portugal; mbernardes09@gmail.com; 10 Department of Rheumatology, São João Hospital Centre EPE, 4200-319 Porto, Portugal; 11 Administração Regional de Saúde do Norte, 4000-477 Porto, Portugal

**Keywords:** bone mineral density, cancellous bone, cortical bone, fructose, mineral water, bone microstructure, ovariectomy

## Abstract

Bone mineral density (BMD) and microstructure depend on estrogens and diet. We assessed the impact of natural mineral-rich water ingestion on distal femur of fructose-fed estrogen-deficient female Sprague Dawley rats. Ovariectomized rats drank tap or mineral-rich waters, with or without 10%-fructose, for 10 weeks. A sham-operated group drinking tap water was included (*n* = 6/group). Cancellous and cortical bone compartments were analyzed by microcomputed tomography. Circulating bone metabolism markers were measured by enzyme immunoassay/enzyme-linked immunosorbent assay or multiplex bead assay. Ovariectomy significantly worsened cancellous but not cortical bone, significantly increased circulating degradation products from C-terminal telopeptides of type I collagen and receptor activator of nuclear factor-kappaB ligand (RANKL), and significantly decreased circulating osteoprotegerin and osteoprotegerin/RANKL ratio. In ovariectomized rats, in cancellous bone, significant water effect was observed for all microstructural properties, except for the degree of anisotropy, and BMD (neither a significant fructose effect nor a significant interaction between water and fructose ingestion effects were observed). In cortical bone, it was observed a significant (a) water effect for medullary volume and cortical endosteal perimeter; (b) fructose effect for cortical thickness, medullary volume, cross-sectional thickness and cortical endosteal and periosteal perimeters; and (c) interaction effect for mean eccentricity. In blood, significant fructose and interaction effects were found for osteoprotegerin (no significant water effect was seen). For the first time in ovariectomized rats, the positive modulation of cortical but not of cancellous bone by fructose ingestion and of both bone locations by natural mineral-rich water ingestion is described.

## 1. Introduction 

Osteoporosis leads to reduced bone mass and strength as well as disrupted bone microarchitecture and, consequently, skeletal fragility, which culminates in an increased risk of bone fracture. Volumetric bone mineral density (vBMD) does not show differences in bone material composition and structural design, and so accounts for only 60% of the variation in bone fragility [1]. Imbalance of remodelling in cancellous and cortical bone compartments, leading to the loss of bone tissue and alteration of bone microarchitecture, can occur as a consequence of differential modulation by estrogen deprivation and aging [1,2,3,4,5]. Estrogen signaling has a prime role in bone growth, maturation, architecture, and turnover [2,6,7,8,9], thus explaining why osteoporosis develops after human menopause and animal ovariectomy. Postmenopausal osteoporosis is the most common metabolic bone disorder [2,6,10,11]. 

Bisphosphonates, such as alendronate, are first-line drug therapy for the primary prevention of osteoporosis, notwithstanding the strong bone beneficial effects of hormone therapy (HT). However, both therapies can have significant and harmful side effects and/or contraindications [6,10]. Comprehensibly, the implementation of life-style interventions for reducing or even preventing postmenopause-associated risk of osteoporosis, either alone or as adjunct of osteoporosis pharmacological therapies, lowering the dosage and treatment time needed, is justified. Among the possible life-style interventions, the change and/or re-education of dietary patterns could be the most relevant due to the diverse range of beneficial effects that comes with it and the low economic burden for its implementation. In fact, Mediterranean diet has been proposed as a modifiable environmental factor relevant for preservation of bone health and prevention of osteoporosis [12]. In contrast, Western diet may be a risk factor for impaired bone metabolism and osteoporosis, through excess acid supply and mineral and vitamin deficiencies [9,13,14], that, if present, may aggravate postmenopausal-related osteoporosis. Interestingly, ingestion of natural mineral-rich waters has been reported to be beneficial for bone health and against postmenopausal- and postovariectomy-related osteoporosis, owing to their specific composition and alkali load [9,13,15,16,17,18,19,20,21,22,23,24]. On the other hand, ingestion of mineral-poor water disturbs bone development, metabolism, and biochemical properties, weakening biomechanical and mineral bone properties in female rats [25]. In line, the consumption of very low mineral water has been linked to osteoblast inhibition, bone resorption activation, and bone mineral reduction, as well as height development retardation in children [26].

Osteoporosis and metabolic dysfunction share similar risk factors [27]. High chronic intake of food and beverages sweetened with fructose, sucrose, or fructose corn syrup (high-fructose diet, prolonged in time) prompts obesity and metabolic changes comparable to what is observed in postmenopause, as demonstrated in rodent experimental models and in humans (reviewed in [28,29,30]). To our knowledge, only very few (and somehow inconsistent) data exist regarding the consequences of high-fructose diet upon bone health in pre and postmenopausal or postovariectomy periods [31,32,33,34,35,36,37,38]. As nowadays fructose is highly consumed worldwide [29,39], it is crucial to fully, and without doubt, uncover its effects upon bone health. Additionally, their modulation by mineral water ingestion has not been published. 

We have (a) previously shown the beneficial metabolic impact of the ingestion of a Portuguese natural mineral-rich water in fructose-fed male and ovariectomized female caesarean-derived (CD) Sprague Dawley rats [28,40,41,42,43,44,45] and (b) recently reviewed the beneficial modulation of metabolic dysfunction features by mineral water consumption [46]. So, this work aimed to evaluate the effects of the ingestion of the abovementioned Portuguese natural mineral-rich water on the consequences of ovariectomy alone or combined with high-fructose consumption, in the distal femur of female CD Sprague Dawley rats. We analyzed bone microstructural properties and vBMD by microcomputed tomography (micro-CT) of both cancellous and cortical bone compartments, along with circulating markers of bone metabolism. 

## 2. Material and Methods 

### 2.1. In Vivo Animal Experimentation and Treatments

Female CD Sprague Dawley rats were bilaterally ovariectomized (*n* = 24) or sham-operated (*n* = 6) (Charles River Laboratories, Châtillon-sur-Chalaronne, France). All animals had the same age at the time of the surgical procedures (13 weeks [47]) to avoid potential fluctuations related to aging in the parameters here evaluated. After arrival at the Animal Facility of the Faculty of Medicine, University of Porto (Porto, Portugal), the animals were acclimatized for 10 days (housed 2 per cage, in an enriched environment) before the beginning of the 10-week experimental protocol, as previously described in [28]. The handling and care of the animals were conducted in conformity with the European Community Council guidelines for the use of experimental animals (86/609/EEC and 2010/63/UE) and Portuguese Acts (129/92 and 113/2013). The animal protocol described below was approved by Direção-Geral de Alimentação e Veterinária (DGAV), the Portuguese Competent Authority for animal research. Ovariectomized rats were randomly distributed into 4 groups (6 animals each) with *ad libitum* access to standard laboratory pellet food (Rodent Maintenance Diet from Harlan Interfauna Iberica S.A., Barcelona, Spain) and distinct drinking solutions (with and without fructose): (a) tap water (TWO; total mineralization lower than 200 mg/L), (b) natural mineral-rich water (MWO: hypersaline sodium-rich naturally sparkling mineral water, in conformity with the European Community Council guidelines for natural mineral waters (2009/54/EEC); total mineralization of 2855 mg/L (Table S1)), (c) 10%-fructose in tap water (TWFO), and (d) 10%-fructose in natural mineral-rich water (MWFO) [28,41]. Sham-operated rats, creating the control group, drank tap water (STW). 

There were no significant differences among the four ovariectomized animal groups regarding body weight (BW) - relevant for preventing biased bone parameter results. Data and corresponding statistical analysis of the evolution of BW as well as fructose and food ingestion were previously published ([28]; values of these two last variables are show in Tables S2 and S3, respectively). In all groups, the calcium threshold (2.5 g Ca/kg diet) necessary to produce normal growth in Sprague Dawley rats was exceeded [28,48]. 

Overnight, fasted animals were deeply anesthetized with sodium pentobarbital (80 mg/kg BW) for blood collection by left ventricle cardiac puncture. After obtaining each femur and removing the muscle (with instruments, carefully without damaging the bone), gauze soaked in physiological saline was wrapped around each femur. Serum aliquots were stored at −80 °C and femora at −20 °C, until further use. 

### 2.2. Assessment of Bone Microstructural Properties and Volumetric Bone Mineral Density by Microcomputed Tomography

#### 2.2.1. Scan, Reconstruction, and Image Processing 

The distal region of the femur was scanned in a desktop X-ray microtomograph (Skyscan 1172; Brucker micro-CT NV, Kontich, Belgium) at the facilities of Trabeculae S.L. (Ourense, Spain). Femora were imaged with an X-ray tube voltage of 70 kV and a current of 142 µA, with a nominal resolution of 13 µm and the use of a 1-mm aluminum filter. The rotation angle used was 185°, with a rotation step of 0.4°. Datasets were reconstructed using a modified Feldkamp algorithm, with NRecon software (Bruker micro-CT N.V., Kontich, Belgium; version 1.6.1.7). The obtained sections were converted into binary images using adaptive global thresholding. For the analysis of microstructural parameters of cancellous and cortical bone, two different volumes of interest (VOI) were selected. In the case of cancellous bone, VOI started to 1 mm from the growth plate of the distal femur (taken as reference section) occupying 4 mm in the proximal direction and excluding cortical bone. For analysis of cortical bone, the VOI began at a distance of 7 mm from the growth plate, and it extended 2 mm in the proximal direction, excluding cancellous bone (Figure S1). The selection of the corresponding VOIs, and consequent bone microstructural properties, and vBMD analyses were carried out with commercial software provided with the micro-CT equipment (SkyScan CT-Analyser; version 1.10.0.2) [49]. Bone measurements were obtained by staff who were blinded to the treatment group of the rats.

#### 2.2.2. Microstructural Parameters of Cancellous Bone

Morphometric indices of cancellous bone region were determined using the datasets, integrated over a VOI, using direct 3D morphometry. According to the American Society for Bone and Mineral Research (ASBMR), when reporting micro-CT results, the minimal set of variables that should be used to describe cancellous bone morphometry includes bone volumetric fraction (BV/TV, %) as well as trabecular thickness (Tb.Th, μm), trabecular spacing (Tb.Sp, μm), and trabecular number (Tb.N, mm^−1^) [50]. Aiming to obtain additional information about the cancellous bone microstructure, we evaluated five more cancellous bone variables. 

Both the tissue volume of VOI (TV, mm^3^) and cancellous bone volume (BV, mm^3^) were calculated based on the hexahedral marching cubes volume model of the VOI. Bone volumetric fraction, specific bone surface (BS/BV, mm^−1^), and bone surface density (BS/TV, mm^−1^) were calculated directly. BS/TV is the ratio of surface area to total volume measured, whereas BS/BV is the ratio for the bone surface per given bone volume that gives a measure on how many bone lining cells cover a given volume of bone. The three trabecular associated variables were measured directly on 3D images. Measurements of trabecular thickness were calibrated by scanning and analyzing three aluminum foils with thicknesses of 50, 125, and 250 mm. Structure model index (SMI), which indicates the relative prevalence of rods and plates in the 3D structure, and trabecular bone pattern factor (Tb.Pf, mm^−1^), which shows the connectedness of a cancellous bone structure described by the relation of convex to concave surfaces of the trabeculae, which were also calculated using the direct 3D model. Degree of anisotropy (DA) represents trabecular anisotropy defined as the ratio between the maximal and minimal radius of the mean intercept length [49].

#### 2.2.3. Microstructural Parameters of Cortical Bone

Cortical bone parameters were measured by micro-CT from the values obtained analyzing each individual transverse section (2D analysis) and interpolating these sections, taking into account the space between them (3D analysis). Parameters obtained by 3D analysis included cortical thickness (Ct.Th, µm), cortical volume (Ct.V, mm^3^), and medullary volume (MV, mm^3^). Parameters obtained by 2D analysis included cross-sectional thickness (Cs.Th; µm; an alternative measure of cortical thickness); mean total cross-sectional bone area (B.Ar, mm^2^); cortical endosteal perimeter (Ct.En.Pm, mm); cortical periosteal perimeter (Ct.Pe.Pm, mm); mean polar moment of inertia (polar, MMI, mm^4^), which denotes the resistance to rotation of a cross-section about a chosen axis; and mean eccentricity (Ecc), a parameter that shows the difference between the elongation of the cortical region (generally elliptical in shape) with respect to a circular shape [49]. 

#### 2.2.4. Volumetric Bone Mineral Density

In both VOI, vBMD (mg/cm^3^) was determined by micro-CT by direct comparison with the attenuation coefficients of two hydroxyapatite phantoms with known density (250 and 750 mg/cm^3^) used as patterns. 

### 2.3. Assessment of Circulating Markers of Bone Metabolism

All quantifications were made at the Department of Clinical Pathology of São João Hospital Centre EPE, Porto, Portugal. Circulating concentrations of N-terminal propeptide of type I procollagen (PINP; AC-33F1 from Immunodiagnostic Systems Limited, Tyne & Wear, United Kingdom), osteopontin (OPN; JP27360 from Immuno-Biological Laboratories, Co. Limited, Gunma, Japan), osteocalcin (OTN; AC-12F1 from Immunodiagnostic Systems Limited, Tyne & Wear, United Kingdom), and degradation products from C-terminal telopeptides of type I collagen (RatLapsTM, CTX-1; AC-06F1 from Immunodiagnostic Systems Limited, Tyne & Wear, United Kingdom) were evaluated by using enzyme immunoassay/enzyme-linked immunosorbent assay (EIA/ELISA), according to the instructions provided by the suppliers. Circulating concentrations of receptor activator of nuclear factor-kappaB ligand (RANKL; RRNKLMAG-31K-01, Single Plex) and osteoprotegerin (OPG; RBN1MAG-31K, two plex) were determined by a plex bead assay performed according to protocols (MILLIPLEX^®^ MAP kits) of Millipore Corporation (Billerica, MA, USA). Quantifications were performed using a Luminex 200 analyzer (Luminex Corporation, Austin, TX, USA).

### 2.4. Statistical Analysis

At the end of the dietary intervention, two-way analysis of variance (ANOVA), followed by Tukey multiple comparison test, was used for the estimation of the effects upon all variables evaluated in the four ovariectomized rat groups. Student’s *t*-test was used to evaluate the effects of ovariectomy upon the same variables. Correlations between circulating markers of bone metabolism and vBMD were calculated using a Spearman two-tailed test (GraphPad Prism software^®^, La Jolla, CA, USA; version 8.1.2). *p* ≤ 0.05 was considered statistically significant (a tendency was considered whenever 0.05 < *p <* 0.1). Results are presented as mean ± standard deviation after mild outlier rejection (1.5 times the interquartile range below the first quartile or 1.5 times the interquartile range above the third quartile). In Tables S2 and S3, the results are presented as mean ± standard error of the mean, according to [28]. 

## 3. Results 

Average BW at the onset of the dietary intervention was 282.2 ± 40.48 g, 330.2 ±3 7.53 g, 335.8 ± 48.71 g, 323.7 ± 41.62 g, 322.2 ± 21.43 g for STW, TWO, MWO, TWFO and MWFO groups, respectively. BW at the end of the dietary intervention was 336.2 ± 72.93 g, 414.8 ± 49.04 g, 413.2 ± 69.88 g, 400.0 ± 77.94 g, and 379.3 ± 28.30 g for STW, TWO, MWO, TWFO, and MWFO groups, respectively. The evolution of BW (g) and food ingestion (g/cage) as well as BW (g/day) and food ingestion (g/cage/day) gains with time, in addition to differences in fructose solution consumption (mL/cage) between TWFO and MWFO groups, along the 10-week experimental protocol, and the values of absolute BW gain (g) have been published elsewhere along with their statistical analysis [28]. Regarding this latter anthropometric parameter, although some variation was observed among groups, no statistical significance (or tendency) was reached. Overall, MWFO and TWFO rats ingested a similar quantity of fructose (Table S2). Over time, (a) MWFO, MWO, and TWFO had significantly lower food ingestion vs. STW, and (b) the decrease of food consumption was significantly higher in TWFO vs. TWO and MWFO vs. MWO. A similar behavior was found in MWO vs. TWO (Table S3) [28].

### 3.1. Microstructural Properties and Volumetric Bone Mineral Density by Micro-CT 

#### 3.1.1. Cancellous Bone Compartment 

**Figure 1:**
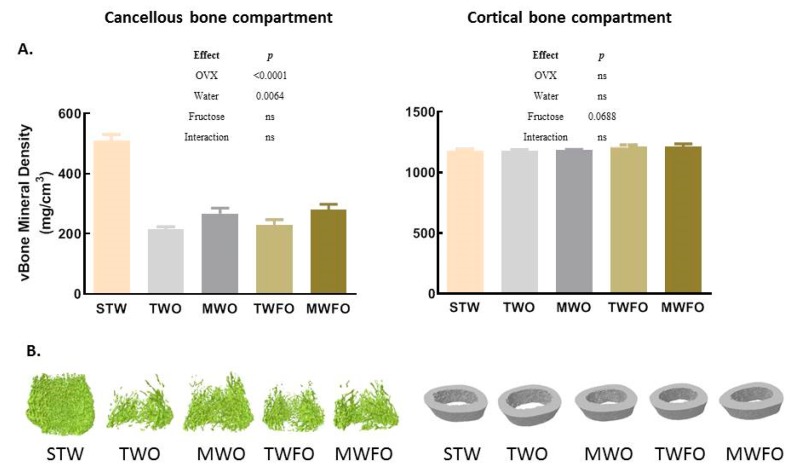
Volumetric bone mineral density of rat cancellous and cortical compartments of distal femur by microcomputed tomography. (**A**) Quantification of volumetric bone mineral density (mg/cm^3^). (**B**) Representative images of the compartments used for quantification of volumetric bone mineral density. MWFO: ovariectomized caesarean-derived (CD) Sprague Dawley rats with access to 10% fructose in natural mineral-rich water; MWO: ovariectomized CD Sprague Dawley rats with access to natural mineral-rich water; ns: nonsignificant; OVX: ovariectomy; STW: sham-operated CD Sprague Dawley rats with access to tap water; TWFO: ovariectomized CD Sprague Dawley rats with access to 10% fructose in tap water; TWO: ovariectomized CD Sprague Dawley rats with access to tap water; vBone: volumetric bone. Results are presented as mean ± standard deviation (after mild outlier rejection: 5 ≤ *n* ≤ 6). Two-way analysis of variance (ANOVA), followed by Tukey multiple comparison test, was used for the estimation of the effects upon volumetric bone mineral density evaluated in the four ovariectomized rat groups. Student’s *t*-test was used to evaluate the effects of ovariectomy upon this variable.

Ovariectomy (TWO vs. STW) significantly modulated all measured microstructural properties of the cancellous bone compartment as well as cancellous vBMD, with the exception for the degree of anisotropy (Table 1 and Figure 1A). 

Ovariectomy significantly decreased bone volumetric fraction (*p <* 0.0001), bone surface density (*p <* 0.0001), trabecular thickness (*p =* 0.0239), trabecular number (*p <* 0.0001), and vBMD (*p <* 0.0001) (Table 1 and Figure 1A). Ovariectomy significantly increased structural model index (*p =* 0.0005; denoting a reduction of plate-like trabeculae), trabecular bone pattern factor (*p =* 0.0006; showing an increase of convex surfaces, indicating a badly connected trabecular lattice), trabecular spacing (*p <* 0.0001), and specific bone surface (*p =* 0.0185) (an increasing pattern was observed for the degree of anisotropy (*p =* 0.0656)) (Table 1). 

A significant water effect in OVX rats was observed for all measured microstructural properties of the cancellous bone compartment as well as cancellous vBMD, with degree of anisotropy being the exception (Table 1 and Figure 1A). In particular, natural mineral-rich water ingestion by OVX rats significantly increased trabecular number, with (TWFO vs. MWFO *p* = 0.0298) and without (TWO vs. MWO *p* = 0.0024) associated fructose consumption; bone surface density, with (*p* = 0.0465) and without (*p* = 0.0055) fructose; and bone volumetric fraction, with (*p* = 0.0138) and without (*p* = 0.0009) fructose, as well as significantly decreased structural model index without fructose (*p* = 0.0101) (a decreasing pattern was observed for trabecular bone pattern factor without fructose (*p* = 0.0735); Table 1). 

In OVX rats, neither a significant fructose effect nor a significant interaction between water and fructose ingestion effects was observed for all measured microstructural properties of the cancellous bone compartment as well as cancellous vBMD (Table 1 and Figure 1A).

#### 3.1.2. Cortical Bone Compartment

No significant effect of ovariectomy (TWO vs. STW) was observed within the microstructural parameters or vBMD of the cortical bone compartment; nevertheless, a tendency for an increase was observed for cortical endosteal perimeter (*p* = 0.0799) (Table 2 and Figure 1A). 

A significant water effect was observed in OVX rats only for the medullary volume and cortical endosteal perimeter (Table 2 and Figure 1A). In particular, natural mineral-rich water ingestion by OVX rats, without associated fructose consumption, significantly reduced medullary volume (*p* = 0.0150) (Table 2). This could be explained by the differences observed between the cortical endosteal (*p* = 0.0704) and periosteal perimeters of TWO vs. MWO groups (a tendency for a similar water effect was also observed for cortical periosteal perimeter (*p* = 0.0708)) (Table 2).

In OVX rats, a significant fructose effect was observed for cortical thickness, medullary volume, cross-sectional thickness, and cortical endosteal and periosteal perimeters; nevertheless, a tendency for a fructose effect was observed for vBMD (*p* = 0.0688) (Table 2 and Figure 1A). In particular, the ingestion of fructose in tap water by OVX rats (TWO vs. TWFO) significantly increased both measures of cortical thickness (Ct.Th (*p* = 0.0318) and Cs.Th (*p* = 0.0101)), whereas significantly decreased medullary volume (*p* = 0.0195) and cortical endosteal perimeter (*p* = 0.0419) (Table 2). The ingestion of fructose in natural mineral-rich water by OVX rats (MWO vs. MWFO) tended to increase mean eccentricity (*p* = 0.0738) (Table 2). Higher eccentricity means generally elongated objects, while decreased values means an approach toward circular shape.

A significant interaction effect was observed only for mean eccentricity (*p* = 0.0346; Table 2), with 18.08% of the total variation of this variable in OVX rats being explained by the interaction between water and fructose intake effects.

### 3.2. Circulating Markers of Bone Metabolism

When comparing TWO vs. STW rats, it can be observed that ovariectomy significantly decreased the levels of OPG (*p* = 0.0415) and the ratio of OPG to RANKL (*p =* 0.0020), marginally increased OTN (*p =* 0.0541), and significantly increased collagen degradation products (*p =* 0.0090) and RANKL (*p =* 0.0003) (Table 3). 

No significant water effect was observed in OVX rats for all circulating markers of bone metabolism; nevertheless, a tendency for a water effect was observed for OTN (*p* = 0.0779) (Table 3).

A significant fructose effect and a significant interaction between water and fructose intake effects were found in OVX rats for OPG (*p* = 0.0160 and *p* = 0.0076, respectively), a tendency for a fructose effect was observed for RANKL (*p* = 0.0780), and a tendency for an interaction between water and fructose intake effects was observed for OPN (*p* = 0.0738). In detail, fructose in tap water significantly increased OPG (*p* = 0.0049) (Table 3), and 30.46% of the total variation of this variable in OVX rats is explained by the interaction between water and fructose intake effects.

## 4. Discussion 

We report distinct effects of dietary and estrogen deprivation interventions on cancellous and cortical bone compartments from distal femur, a concept consistent with previous results [4,5,36,51,52]. To our knowledge, we are the first to describe differential effects of fructose-feeding within an estrogen-deficiency environment, on cancellous and cortical bone compartments, as well as to characterize their modulation by natural mineral-rich water consumption.

For cancellous bone compartment parameters, except for the degree of anisotropy, a significant water effect was observed; although not always in a statistically significant way, natural mineral-rich water ingestion, with and without fructose consumption, consistently decreased ovariectomy effects. The increases in bone volumetric fraction, bone surface density, trabecular thickness, and trabecular number as well as decreases in specific bone surface, trabecular spacing, trabecular bone pattern factor, and structural model index seem to suggest a decreased cancellous bone loss, producing also changes in trabecular vBMD.

Previously, we have shown that the intake of the same Portuguese natural mineral-rich water originated a similar pattern on several metabolic and redox parameters as well as signaling pathways on male fructose-fed CD Sprague Dawley rats [40,41,42]. Remarkably, we have also described that the intake of this natural mineral-rich water induces signaling through Sirt1/p-AMPK/PGC1α in male and ovariectomized female CD Sprague Dawley rats, fructose-fed or not [28,42,43], which has a beneficial impact upon bone and cartilage and protects against osteoporosis [53,54]. 

### 4.1. Ovariectomy

At the time of ovariectomy and dietary protocol implementation, the female CD Sprague Dawley rats had not reached peak bone mass, leaving some potential for skeletal growth [55]. As cancellous bone loss is higher soon after estrogen deficiency happens, whereas cortical bone loss accelerates later after estrogen deficiency [1,3,4,5], our animal ovariectomy protocol is validated as an inducer of postmenopausal osteoporosis [11]. Nevertheless, it should be referred that estrogen-deficiency modulation of cortical bone might be species- and genetics-dependent, as an uniform reduction of cortical tibia area without alteration of average cortical thickness, along with smaller total tissue area and larger medullar cavity in a non-site-specific manner, was observed 10 weeks after ovariectomy in 8-week old C57Bl/6 mice. Additionally, site-specific modulation of eccentricity and polar moment of inertia was observed [56].

We observed that ovariectomy had a significant effect upon cancellous but not upon cortical bone compartment. The worsening of the cancellous bone was revealed by the significant increase of specific bone surface, trabecular spacing, trabecular bone pattern factor (indicative of reduced trabecular connectivity) and structural model index (reflecting increased rod-like trabecular geometry) [36] as well as significant decrease of bone volumetric fraction, trabecular thickness and number, and bone surface density besides vBMD. Overall, as expected, these results highlight compromised structural integrity and indicate a higher degree of anisotropy in cancellous bone [35,36]. Indeed, we have found a 47% nonsignificant increase in this last variable.

As predicted, ovariectomy significantly decreased circulating OPG and OPG/RANKL ratio, marginally increased OTN, and significantly increased RANKL and CTX-1. OPG and RANKL have opposite roles against bone loss [5,7,57]. These alterations in the circulating bone markers concur with the changes found in cancellous bone microarchitecture and vBMD. Indeed, significantly positive and negative correlations were found between OPG/RANKL ratio (*r* = 0.7697, *p*(two-tailed) = 0.0126), RANKL (*r* = −0.8333, *p*(two-tailed) = 0.0083), and circulating CTX-1 (*r* = −0.6333, *p*(two-tailed) = 0.0407) vs. cancellous bone vBMD, when considering STW and TWO rats. Furthermore, in accordance with findings in postmenopausal women in the presence or absence of osteopenia, mild osteoporosis and severe osteoporosis [8,58], we observed a strong tendency for a negative correlation between circulating OTN and cancellous bone vBMD when considering, as before, STW and TWO rats (*r* = −0.5734, *p*(two-tailed) = 0.0556). In fact, decreased levels of hydroxyapatite crystals in osteoporosis lead to the release of OTN into the blood [8] what may explain the correlation described above.

### 4.2. Fructose

In the cortical bone compartments of OVX rats, a significant fructose effect was observed for cortical thickness, medullary volume, cross-sectional thickness, and cortical endosteal and periosteal perimeters; in blood, it was also seen for OPG. No fructose effect was found for cancellous bone. The ingestion of fructose in tap water by OVX rats significantly increased circulating OPG and cortical and cross-sectional thickness and significantly decreased cortical endosteal perimeter and medullary volume. Interestingly, taking into consideration previously published results on cortical bone parameters and fracture risk, the fructose-induced cortical bone thickening (increased circulating OPG most probably reflecting decreased cortical bone resorption) could contribute to a lower fracture risk in TWFO [7,57,59,60,61,62]. As an example, cortical femur thinning has been shown to identify 21% additional fracture cases over the 57% identified by a T-score < −2.5 [60].

Beneficial and/or harmful, as well as an absence, of effects have been reported concerning fructose-feeding impact on bone health, where differences in animal species, strain, sex, hormonal environment, and protocol of fructose dietary intervention (administration as drinking solution or pellet food, and duration time) may explain the differences observed [31,32,33,34,35,36,37,38]. To our knowledge, this is the first report of a beneficial specific effect of fructose feeding upon cortical bone in an estrogen-deficient background. Likewise, Hanayama evaluated the effect of fructose-feeding upon osteoporosis, by using ovariectomized female Wistar rats (10 weeks old) fed a 60% fructose diet, for 28 days. However, differently from us, they observed a significant increase in osteoclast activity in trabecular bone at the proximal tibia, a significant decrease of tibia and femur BMD as well as a significant increase of urinary deoxypyridinoline of ovariectomized fructose-fed rats vs. ovariectomized control rats [31].

### 4.3. Natural Mineral-Rich Water

A significant water effect was observed for both cortical (for medullary volume and cortical endosteal perimeter) and cancellous (for all parameters, but the degree of anisotropy) bone, but not for blood. In cancellous bone, we found that the aforesaid overall apparent pattern of natural mineral-rich water ingestion prevention against ovariectomy detrimental effects was more evident for trabecular number, bone surface density, and bone volumetric fraction, with significant differences being obtained with and without fructose, followed by trabecular bone pattern factor and structural model index with a tendency and significant difference, respectively, being perceived without fructose. A moderate prevention of bone loss with the ingestion of mineral-rich water was observed. Interestingly, the majority of the statistical relevant effects of the intake of the natural mineral-rich water tested here occurred in the group with higher mineral intake from food (fructose-fed rats voluntarily ate less food than rats without access to fructose, independently of the water type) [28]. Although, the calcium content in our natural mineral-rich water is much lower than the calcium content values of waters used in other published studies [16,19], the high bicarbonate content in our natural mineral-rich water should have reduced renal calcium excretion [46,63] and ameliorated the calcium balance (a similar modulation should have occurred for magnesium [46,64]) further contributing to/explaining the beneficial impact observed on the distal femur. 

Our advantageous results upon cortical and cancellous bone compartment microstructural properties variables are consistent with previous favorable findings upon distinct bone-related parameters, taking into consideration natural mineral-rich water consumption by postmenopausal women [15,16,18,19] and ovariectomized rats [22]. Bearing in mind that the natural mineral-rich water used here has a high bicarbonate content, Roux reported that bone resorption markers, namely, urinary CTX-1, total pyridinoline, and deoxypyridinoline (corrected to urinary creatinine), were significantly reduced in postmenopausal healthy women drinking a natural mineral water rich in bicarbonate compared to baseline, but were not significantly modified when a natural mineral water rich in sulfates, and a similar high-calcium concentration, was ingested (both 1 L/day, for 28 days). Alkalinization of the urine was observed with the former water, what associates with an improved calcium balance [18]. Cepollaro, studying the effect of high-calcium or low-calcium mineral waters in early postmenopausal women, for 13 months, supported the use of a high-calcium mineral water as an effective prophylaxis against postmenopausal bone loss [15]. In line, Costi disclosed that a higher calcium intake, owing to the regular lifelong consumption of a high-calcium (318 mg/L) natural mineral water vs. low-calcium natural mineral or tap water (120 to 137 mg/L), was a significant predictor of spinal BMD in postmenopausal but not in premenopausal women [16]. Meunier showed that daily consumption, for 6 months, of 1 L of a high-calcium (596 mg/L) natural mineral water lowered serum PTH and indices of bone turnover (OTN and bone alkaline phosphatase in serum and CTX-1 in serum and urine) in postmenopausal women with low-calcium intake (no postmenopausal women with high-calcium intake were included in this evaluation) [19]. Similarly, Ogata, investigating the relationship between high-mineral drinking water intake and osteogenesis in ovariectomized rats submitted to low-calcium diet+purified water or low-calcium diet+high-mineral drinking water (0.0468% Ca in total) for three months, concluded that high-mineral drinking water could be beneficial for maintaining bone quantity. Specifically, bone density was higher in the group ingesting high-mineral drinking water although a decrease in the amount of skeletal calcium was observed, highlighting that high-mineral drinking water contributes to the maintenance of bone density but not to the amount of calcium in bone. High-mineral drinking water also increased circulating and bone magnesium [22]. 

Emphasizing water mineral content relevance in bone health, an inverse association between calcium in drinking water and hip fracture risk was found in men in a Norwegian prospective study [23], and a review showed that alkali mineral waters with a low potential renal acid load value and a high bicarbonate content exert an inhibitory effect on bone resorption that exceeds the effect of mineral waters that are only rich in calcium [21]. 

The results of this study show interesting effects of ingestion of natural mineral-rich water in both bone microstructure and density. 

## 5. Conclusions

The relevance of water composition and pH upon bone health has been highlighted in several reports mentioned in this original article. More contradictory results can be found in the literature regarding fructose effects in bone tissue. Our results showed that although ovariectomy negatively influenced the cancellous but not cortical bone compartment of the distal femur, fructose ingestion by ovariectomized animals positively modulated cortical but not the cancellous bone compartment. Natural mineral-rich water ingestion by itself exerted positive effects upon both bone locations. Almost exclusively, these variations were accompanied by modifications of circulating levels of bone metabolism markers when evaluating ovariectomy. The intake of this Portuguese natural mineral-rich water as well as fructose may prove to be relevant for primary prevention against osteoporosis risk fracture as a complement, or even an alternative, to hormone therapy. In future studies, it would be convenient to (a) include an ovariectomized group with estrogen/hormonal supplementation, (b) include further time points and/or older animals, (c) quantify the bioavailability of the bone-related mineral content of the Portuguese natural mineral-rich water, (d) measure the impact of the consumption of the Portuguese natural mineral-rich water upon acid-base status and bone-related minerals homeostasis, and (e) complement the evaluation done here, with a functional assessment of the bone (such as the three-point bending test) to support, bear out, and explain our results. 

## Figures and Tables

**Table 1:** Cancellous bone region (rat distal femur) evaluation of microstructural properties by microcomputed tomography.

	STW	TWO	MWO	TWFO	MWFO	*p* OVX Effect	*p* Water Effect	*p* Fructose Effect	*p* Interaction
**Bone volumetric fraction (BV/TV, %)**	30.63 ± 6.69	6.23 ± 0.37	11.33 ± 2.83	7.07 ± 1.41	10.65 ± 1.50	<0.0001	<0.0001 (TWO vs. MWO 0.0009; TWFO vs. MWFO 0.0138)	ns	ns
**Specific bone surface (BS/BV, mm^−1^)**	34.53 ± 3.81	41.39 ± 4.61	36.70 ± 3.02	39.30 ± 4.37	37.25 ± 1.36	0.0185	0.0405	ns	ns
**Bone surface density (BS/TV, mm^−1^)**	10.37 ± 1.30	2.62 ± 0.19	4.09 ± 0.73	2.85 ± 0.75	3.89 ± 0.63	<0.0001	0.0001 (TWO vs. MWO 0.0055; TWFO vs. MWFO 0.0465)	ns	ns
**Trabecular thickness (Tb.Th, µm)**	110.91 ± 6.61	99.82 ± 7.78	107.77 ± 7.46	100.13 ± 5.62	106.37 ± 2.36	0.0239	0.0181	ns	ns
**Trabecular spacing (Tb.Sp, µm)**	213.32 ± 29.88	972.04 ± 173.12	807.39 ± 170.42	1046.67 ± 247.39	838.22 ± 64.15	<0.0001	0.0230	ns	ns
**Trabecular number (Tb.N, mm^−1^)**	2.74 ± 0.46	0.63 ± 0.03	1.04 ± 0.19	0.70 ± 0.18	0.98 ± 0.16	<0.0001	<0.0001 (TWO vs. MWO 0.0024; TWFO vs. MWFO 0.0298)	ns	ns
**Trabecular bone pattern factor (Tb.Pf, mm^−1^)**	6.05 ± 3.94	15.73 ± 2.74	12.81 ± 1.07	14.60 ± 1.94	12.85 ± 0.86	0.0006	0.0069 (TWO vs. MWO 0.0735)	ns	ns
**Structural model índex** **(SMI)**	1.43 ± 0.44	2.38 ± 0.15	2.13 ± 0.13	2.31 ± 0.07	2.20 ± 0.12	0.0005	0.0016 (TWO vs. MWO 0.0101)	ns	ns
**Degree of anisotropy** **(DA)**	0.34 ± 0.14	0.50 ± 0.14	0.43 ± 0.04	0.45 ± 0.17	0.46 ± 0.11	0.0656	ns	ns	ns

MWFO: ovariectomized caesarean-derived (CD) Sprague Dawley rats with access to 10% fructose in natural mineral-rich water; MWO: ovariectomized CD Sprague Dawley rats with access to natural mineral-rich water; ns: nonsignificant; OVX: ovariectomy; STW: sham-operated CD Sprague Dawley rats with access to tap water; TWFO: ovariectomized CD Sprague Dawley rats with access to 10% fructose in tap water; TWO: ovariectomized CD Sprague Dawley rats with access to tap water. Results are presented as mean ± standard deviation (after mild outlier rejection: 5 ≤ *n* ≤ 6). Two-way analysis of variance (ANOVA), followed by Tukey multiple comparison test, was used for the estimation of the effects upon all cancellous bone microstructural properties evaluated in the four ovariectomized rat groups. Student’s *t*-test was used to evaluate the effects of ovariectomy upon these variables.

**Table 2:** Cortical bone region (rat distal femur) evaluation of microstructural properties by microcomputed tomography.

	STW	TWO	MWO	TWFO	MWFO	*p* OVX Effect	*p* Water Effect	*p* Fructose Effect	*p* Interaction
**Cortical thickness (Ct.Th, µm)**	496.13 ± 43.84	457.67 ± 56.96	489.40 ± 21.65	539.79 ± 61.06	535.48 ± 24.52	ns	ns	0.0049 (TWO vs. TWFO 0.0318)	ns
**Cortical volume** **(Ct.V, mm^3^)**	10.51 ± 0.77	10.92 ± 0.57	11.20 ± 1.15	11.24 ± 1.02	11.29 ± 0.40	ns	ns	ns	ns
**Medullary** **volume (MV, mm^3^)**	7.76 ± 1.54	9.32 ± 1.44	6.93 ± 0.63	7.12 ± 1.52	6.24 ± 0.68	ns	0.0033 (TWO vs. MWO 0.0150)	0.0080 (TWO vs. TWFO 0.0195)	ns
**Cross-sectional thickness (Cs.Th, µm)**	440.96 ± 62.50	404.95 ± 59.33	455.86 ± 42.76	502.25 ± 53.71	497.16 ± 17.56	ns	ns	0.0024 (TWO vs. TWFO 0.0101)	ns
**Mean total cross-sectional bone area** **(B.Ar, mm^2^)**	5.35 ± 0.48	5.46 ± 0.48	5.45 ± 0.54	5.50 ± 0.48	5.53 ± 0.21	ns	ns	ns	ns
**Cortical periosteal** **perimeter (Ct.Pe.Pm, mm)**	11.27 ± 0.77	11.92 ± 0.44	11.25 ± 0.68	11.18 ± 0.73	10.96 ± 0.08	ns	0.0708	0.0399	ns
**Cortical endosteal perimeter (Ct.En.Pm, mm)**	7.25 ± 0.73	7.99 ± 0.59	7.14 ± 0.47	7.06 ± 0.76	6.62 ± 0.20	0.0799	0.0126 (TWO vs. MWO 0.0704)	0.0058 (TWO vs. TWFO 0.0419)	ns
**Mean polar moment of inertia (polar, MMI, mm^4^)**	11.97 ± 2.64	13.96 ± 1.93	12.08 ± 2.52	11.99 ± 2.82	11.12 ± 0.34	ns	ns	ns	ns
**Mean eccentricity** **(Ecc)**	0.75 ± 0.03	0.74 ± 0.02	0.73 ± 0.04	0.73 ± 0.02	0.77 ± 0.03	ns	ns	ns (MWO vs. MWFO 0.0738)	0.0346

MWFO: ovariectomized caesarean-derived (CD) Sprague Dawley rats with access to 10% fructose in natural mineral-rich water; MWO: ovariectomized CD Sprague Dawley rats with access to natural mineral-rich water; ns: nonsignificant; OVX: ovariectomy; STW: sham-operated CD Sprague Dawley rats with access to tap water; TWFO: ovariectomized CD Sprague Dawley rats with access to 10% fructose in tap water; TWO: ovariectomized CD Sprague Dawley rats with access to tap water. Results are presented as mean ± standard deviation (after mild outlier rejection: 5 ≤ *n* ≤ 6). Two-way analysis of variance (ANOVA), followed by Tukey multiple comparison test, was used for the estimation of the effects upon all cortical bone microstructural properties evaluated in the four ovariectomized rat groups. Student’s *t*-test was used to evaluate the effects of ovariectomy upon these variables.

**Table 3:** Circulating markers of bone metabolism.

	STW	TWO	MWO	TWFO	MWFO	*p* OVX Effect	*p* Water Effect	*p* Fructose Effect	*p* Interaction
**N-terminal propeptide of type I procollagen (ng/mL)**	6.87 ± 4.40	5.75 ± 3.88	4.24 ± 1.78	3.33 ± 2.87	3.61 ± 2.59	ns	ns	ns	ns
**Osteopontin (ng/mL)**	40.32 ± 7.02	41.98 ± 5.48	40.41 ± 1.87	35.90 ± 5.11	41.21 ± 2.72	ns	ns	ns (TWO vs. TWFO 0.0959)	0.0738
**Osteocalcin (ng/mL)**	156.73 ± 43.53	204.14 ± 30.74	169.30 ± 17.07	175.18 ± 3.76	172.50 ± 24.97	0.0541	0.0779 (TWO vs. MWO 0.0935)	ns	ns
**CTX-1 (ng/mL)**	16.27 ± 4.47	24.23 ± 3.21	22.90 ± 6.76	22.09 ± 5.60	23.74 ± 0.60	0.0090	ns	ns	ns
**OPG (pg/mL)**	280.24 ± 54.54	208.72 ± 17.61	243.63 ± 18.16	286.51 ± 46.01	238.64 ± 9.56	0.0415	ns (TWFO vs. MWFO 0.0954)	0.0160 (TWO vs. TWFO 0.0049)	0.0076
**RANKL (pg/mL)**	13.15 ± 0.79	17.04 ± 0.95	16.10 ± 3.32	22.15 ± 1.25	17.93 ± 6.33	0.0003	ns	0.0780	ns
**OPG/RANKL**	21.38 ± 4.29	12.40 ± 1.28	13.91 ± 3.87	11.82 ± 0.86	15.05 ± 4.94	0.0020	ns	ns	ns

CTX-1: degradation products from C-terminal telopeptides of type I collagen; MWFO: ovariectomized caesarean-derived (CD) Sprague Dawley rats with access to 10% fructose in natural mineral-rich water; MWO: ovariectomized CD Sprague Dawley rats with access to natural mineral-rich water; ns: nonsignificant; OPG: osteoprotegerin; RANKL: Receptor activator of nuclear factor kappa-Β ligand; OVX: ovariectomy; STW: sham-operated CD Sprague Dawley rats with access to tap water; TWFO: ovariectomized CD Sprague Dawley rats with access to 10% fructose in tap water; TWO: ovariectomized CD Sprague Dawley rats with access to tap water. Results are presented as mean ± standard deviation (after mild outlier rejection: 4 ≤ *n* ≤ 6). Two-way analysis of variance (ANOVA), followed by Tukey multiple comparison test, was used for the estimation of the effects upon all circulating markers of bone metabolism evaluated in the four ovariectomized rat groups. Student’s *t*-test was used to evaluate the effects of ovariectomy upon these parameters.

## Supplementary Materials

The following are available online at https://www.mdpi.com/2072-6643/11/10/2316/s1, Figure S1: Representation of the compartments used for the analysis of cancellous and cortical bone in rat distal femur. Table S1: Chemical characteristics of the Portuguese natural mineral-rich water. Table S2: Fructose solution ingestion values for the 2 groups of rats treated with 10% fructose in the drinking solution along the 10-week intervention period. Table S3: Food ingestion values for all 5 groups of rats along the 10-week intervention period.
