# Supplementary material for: Differential Modulation of Cancellous and Cortical Distal Femur by Fructose and Natural Mineral-Rich Water Consumption in Ovariectomized Female Sprague Dawley Rats
Source: Nutrients. 2019 Sep 30;11(10):2316. doi: 10.3390/nu11102316 (PMC6835992; doi:10.3390/nu11102316)
Supplement: Supplementary file 1 [file nutrients-11-02316-s001.pdf]

Table 1. Chemical characteristics of the Portuguese natural mineral-rich water.

Characteristics	Hypersaline sodium-rich naturally sparkling mineral water
pH	6.16
Total mineralization (mg/L)	2855
Sodium (mg/L)	591
Calcium (mg/L)	92.5
Magnesium (mg/L)	26.2
Potassium (mg/L)	29.9
Copper (mg/L)	0.0013
Zinc (μg/L)	1.1
Selenium (μg/L)	<2.0
Bicarbonate (mg/L)	2013
Chloride (mg/L)	30.8
Sulphate (mg/L)	6.4

Table 2. Fructose solution ingestion values for the 2 groups of rats treated with 10% fructose in the drinking solution along the 10-week intervention period.

	Fructose solution ingestion (mL/cage)			
**Week**	**TWFO**		**MWFO**	
0	126.0	9.4	150.0	19.4
1	311.1	31.0	370.6	50.2
2	259.1	37.7	271.7	46.2
3	303.3	28.7	310.6	38.5
4	295.0	22.2	271.1	32.7
5	283.3	31.2	315.6	33.0
6	272.8	29.5	307.8	32.8
7	281.1	41.4	266.1	14.2
8	243.3	25.6	281.1	32.6
9	236.1	20.4	265.0	29.4
10	282.3	21.6	383.3	69.5

MWFO, ovariectomized caesarean-derived (CD) Sprague-Dawley rats with access to 10% fructose in natural mineral-rich water; TWFO, ovariectomized CD Sprague-Dawley rats with access to 10% fructose in tap water; Results are presented as mean±SEM. Their statistical analysis has been published in Das, J.K., Severo, M., Pereira, C.D., Patricio, E., Magalhães, J., Monteiro, R., Neves, D., Martins, M.J. Natural mineral-rich water ingestion by ovariectomized fructose-fed Sprague-Dawley rats: effects on sirtuin 1 and glucocorticoid signaling pathways. Menopause. 2017;24(5):563-73.

Table 3. Food ingestion values for all 5 groups of rats along the 10-week intervention period.

	Food ingestion (g/cage)									
**Week**	**STW**		**TWO**		**MWO**		**TWFO**		**MWFO**	
**0**	**290.0**	**16.2**	354.3	9.4	392.7	12.7	333.7	7.8	320.7	16.9
1	286.7	19.5	362.3	6.7	405.7	23.4	284.0	22.5	270.7	17.0
2	298.3	21.4	361.2	13.1	396.5	28.5	270.8	35.9	234.7	21.7
3	291.7	19.9	347.2	4.4	370.2	18.7	236.5	30.0	215.0	12.8
4	279.7	27.4	312.3	3.9	314.3	6.8	201.7	16.9	184.3	10.2
5	239.3	7.9	299.3	8.7	301.0	19.6	183.7	19.0	171.3	6.8
6	282.7	3.9	309.1	16.9	312.2	28.1	200.4	12.8	185.6	10.1
7	281.7	23.7	294.3	14.7	312.3	14.6	139.3	47.8	184.3	11.2
8	304.7	37.9	300.7	13.9	311.7	15.8	195.0	19.6	193.7	14.0
9	276.3	20.1	298.0	15.9	298.7	5.4	204.3	22.7	193.0	21.0
10	267.0	24.0	290.3	20.2	290.3	19.8	196.0	27.5	189.7	15.8

MWFO, ovariectomized caesarean-derived (CD) Sprague-Dawley rats with access to 10% fructose in natural mineral-rich water; MWO, ovariectomized CD Sprague-Dawley rats with access to natural mineral-rich water; ns, non-significant; STW, sham-operated CD Sprague-Dawley rats with access to tap water; TWFO, ovariectomized CD Sprague-Dawley rats with access to 10% fructose in tap water; TWO, ovariectomized CD Sprague-Dawley rats with access to tap water. Results are presented as mean±SEM. Their statistical analysis has been published in Das, J.K., Severo, M., Pereira, C.D., Patricio, E., Magalhães, J., Monteiro, R., Neves, D., Martins, M.J. Natural mineral-rich water ingestion by ovariectomized fructose-fed Sprague-Dawley rats: effects on sirtuin 1 and glucocorticoid signaling pathways. Menopause. 2017;24(5):563-73.

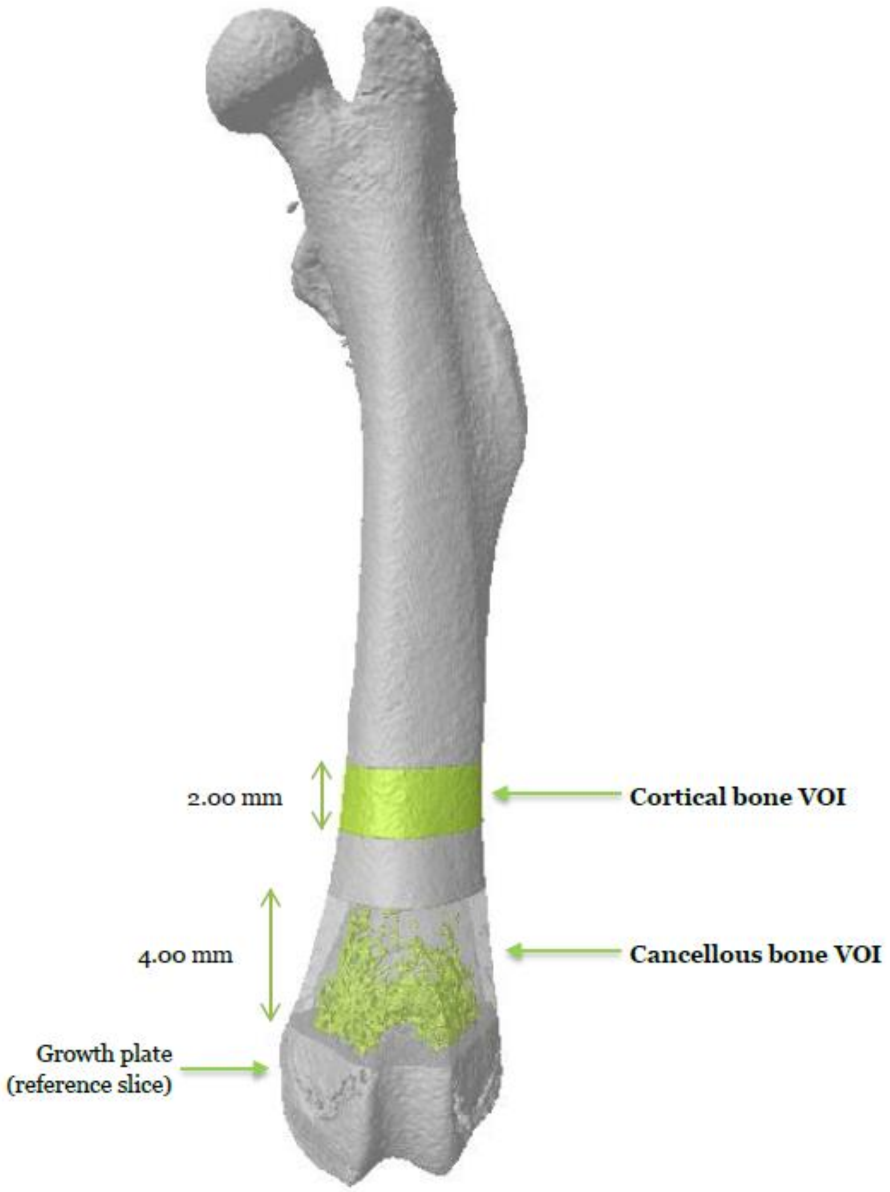

Figure S1. Representation of the compartments used for the analysis of cancellous and cortical bone in rat distal femur. VOI: volume of interest.
